# 

**Published:** 1959-10

**Authors:** 


### Contents

BABY FARMING IN ENGLAND . . . . *Philip Evans* 83

THE USE OF AN ANIMAL LUNG AS OXYGENATOR IN A HEART-LUNG MACHINE . . *M. G. Wilson and o:hers* 90

